# Rejection of the genetic implications of the “Abundant Centre Hypothesis” in marine mussels

**DOI:** 10.1038/s41598-020-57474-0

**Published:** 2020-01-17

**Authors:** Noxolo N. Ntuli, Katy R. Nicastro, Gerardo I. Zardi, Jorge Assis, Christopher D. McQuaid, Peter R. Teske

**Affiliations:** 1 Centre for Ecological Genomics and Wildlife Conservation, Department of Zoology, University of Johannesburg, Auckland Park, 2006 South Africa; 2 CCMAR - Centro de Ciências do Mar, Universidade do Algarve, Faro, Portugal; 3 Department of Zoology and Entomology, Rhodes University, Grahamstown, 6140 South Africa

**Keywords:** Ecology, Genetics, Ocean sciences

## Abstract

The ‘Abundant-Centre Hypothesis’ is a well-established but controversial hypothesis stating that the abundance of a species is highest at the centre of its range and decreases towards the edges, where conditions are unfavourable. As genetic diversity depends on population size, edge populations are expected to show lower intra-population genetic diversity than core populations, while showing high inter-population genetic divergence. Here, the genetic implications of the Abundant-Centre Hypothesis were tested on two coastal mussels from South Africa that disperse by means of planktonic larvae, the native *Perna perna* and the invasive *Mytilus galloprovincialis*. Genetic structure was found within *P*. *perna*, which, together with evidence from Lagrangian particle simulations, points to significant reductions in gene flow between sites. Despite this, the expected diversity pattern between centre and edge populations was not found for either species. We conclude that the genetic predictions of the Abundant-Centre Hypothesis are unlikely to be met by high-dispersal species with large population sizes, and may only become evident in species with much lower levels of connectivity.

## Introduction

The Abundant-Centre Hypothesis (ACH) states that species perform better at the centre of their range, where high densities are observed, and that densities decline towards the range edges^1,2^. Both biotic and abiotic factors determine the success of a species within its realised niche^2^, and it is expected that population abundance, reproductive output and recruitment will decline towards the periphery of its distribution, due to the less favourable conditions prevailing there^2,3^. Patterns of population genetic structure across a species’ range are, in turn, expected to be influenced by these ecological effects. Because genetic diversity depends on population size, it is predicted that populations at the periphery of the species’ range will show lower intra-population diversity and higher inter-population divergence when compared to centre populations^2–4^. These differences are attributed to reduced gene flow, bottleneck effects, habitat fragmentation and random genetic drift in smaller populations^5^. The reduced diversity and increased genetic differentiation of edge populations may translate into lower adaptive potential particularly at the periphery of a species’ range^6,7^, which suggests that maladaptation may cause these populations to be demographic sinks.

Several recent studies have raised doubts about the generality of these demographic and genetic expectations. A review found that there is limited empirical evidence for the ACH, with support from only 39% of the reviewed studies, which covered a wide range of genetic and demographic predictions derived from the hypothesis^8^. Similarly, another review assessing population genetic structure across the geographical ranges of 115 species of plants and animals showed that, on average, only 64% of studies detected a decline in genetic diversity towards the range margins, although there was a geographic bias as the majority of studies concerned the northern range limits of northern hemisphere species^9^. Taken together, these studies show that species abundance, demography and genetic diversity appear to be more variable than predicted by the ACH, as they are determined by the interaction of multiple fluctuating contemporary environmental drivers plus historical effects^9,10^.

Along the South African coastline, rocky shores are a very common habitat type. They constitute nearly 60% of the coastline and span four major biogeographic regions^11,12^, making common rocky shore fauna particularly suitable for studying the ACH. We tested the predictions of the ACH for the dominant rocky shore mussels in this region^11^, the invasive *Mytilus galloprovincialis* and the native *Perna perna*. The latter is represented by two distinct regional genetic lineages^12^, effectively providing edge and centre populations for three model organisms with similar biology. We hypothesised that centre populations of the three mussels would have greater genetic diversity than edge populations and tested this by comparing genetic diversity for the two population types.

## Materials and Methods

### Study species and their ranges

**Figure 1:**
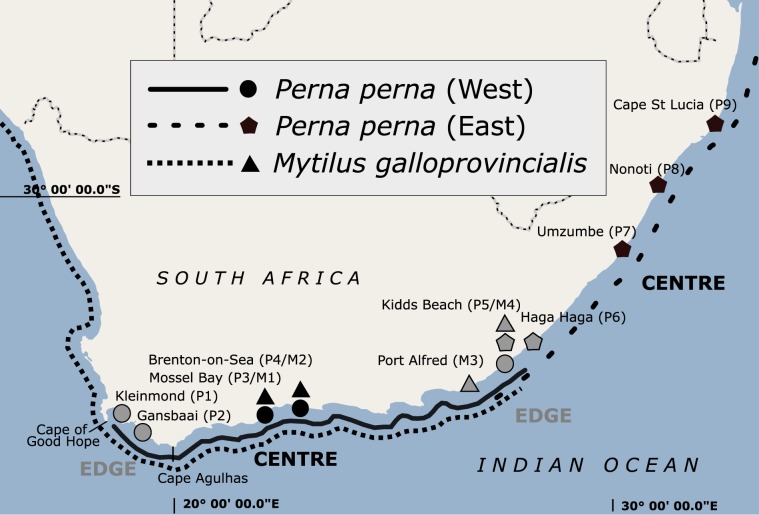
Sampling sites for the western (circles) and eastern (pentangle) mtDNA COI lineages of *Perna perna*, and of *Mytilus galloprovincialis* (triangles). Centre (black) and edge (grey) sites are indicated for each lineage/species. The map was created in StepMap (https://www.stepmap.de/) and modified in InkScape 0.92.4 (https://inkscape.org).

*Perna perna* has a wide range from the warm-temperate south coast of South Africa to the tropical coastline of southern Mozambique on the east coast^11^. Due to the cold upwelled water of the Benguela Current on the west coast, *P*. *perna* does not occur west of the Cape of Good Hope, but it re-emerges in Namibia, and its range stretches along the west coast of Africa all the way to the Mediterranean Sea^13^. Previous genetic work using mitochondrial DNA (mtDNA) sequences and microsatellite loci of *P*. *perna* identified a phylogeographic break on the south-east coast of South Africa, where the ranges of a western and an eastern lineage overlap along 200 km of coastline, from approximately Port Alfred to Haga Haga^14,15^ (Fig. 1). A subsequent study showed a non-sister relationship for the two lineages, possibly reflecting an Indo-Pacific origin followed by dispersal into the Atlantic through the Tethys seaway, independent southward expansion along the western and eastern shores of the African continent and recent secondary contact on the south-east coast of South Africa^16^.

The other dominant mussel in South Africa, the Mediterranean mussel *Mytilus galloprovincialis*, is a particularly successful marine invader that has established itself on all continents except Antarctica^17^. In South Africa, *M*. *galloprovincialis* is found in cooler waters west of the Cape of Good Hope where *P*. *perna* is absent. Both species co-exist on the south coast, while only *P*. *perna* occurs along the subtropical and tropical east coast.

For both the western and eastern lineages of *P*. *perna*, sites on the south-east coast where the ranges of these lineages overlap constitute edge populations, and the same region also constitutes the range periphery of *M*. *galloprovincialis*. On the south-west coast, the western lineage of *P*. *perna* has a second edge population (Fig. 1 and Table 1).

**Table 1:** Sampling sites for *Perna perna* and *Mytilus galloprovincialis*, and assignment as centre or edge populations. Lineage ancestry of *Perna perna* was based on previous studies^14,15,18^.

Species	Lineage	Sampling Sites	Code	GPS Coordinates	N	Site Category
*Perna perna*	West	Kleinmond	P1	−34.33, 19.01	47	edge
	West	Gansbaai	P2	−34.60, 19.34	47	edge
	West	Mossel Bay	P3	−34.19, 22.11	47	centre
	West	Brenton-on-Sea	P4	−34.07, 23.02	45	centre
	Both	Kidd’s Beach	P5	−33.15, 27.70	46	edge
	Both	Haga Haga	P6	−32.76, 28.25	41	edge
	East	Umzumbe	P7	−30.61, 30.55	48	centre
	East	Nonoti	P8	−29.67, 30.66	47	centre
	East	Cape St Lucia	P9	−28.51, 32.41	47	centre
*Mytilus galloprovincialis*		Mossel Bay	M1	−34.19, 22.11	48	centre
		Brenton-on-Sea	M2	−34.07, 23.02	48	centre
		Port Alfred	M3	−33.60, 26.89	48	edge
		Kidd’s Beach	M4	−33.15, 27.70	48	edge

Decimal coordinates represent latitude and longitude, respectively; N: sample size.

Assignment of sites as either centre or edge populations was decided *a priori* based on previous intensive field surveys^14,15,18^. These identified the area between approximately Port Alfred to Haga Haga as being located in a biogeographical transition zone and thus edge habitat, while Cape Agulhas (Fig. 1) was identified as the location that separated core populations on the south coast from western edge populations.

### Estimation of mussel cover

To confirm that pre-defined edge populations had smaller population densities than core populations, percentage mussel cover at specific sites was estimated with ImageJ software^19^ using unpublished data from earlier fieldwork. Mussel cover was measured between January and April 2013 by haphazardly placing quadrats (25 × 25 cm; N = 20) onto mussel beds. Site M3 (Fig. 1) was surveyed in 2011, and for site P8, which is difficult to access, the survey was conducted at a nearby location (Ballito). No data were available for site P9.

Estimates of cover for the two lineages of *P*. *perna* at sites P5 and P6 could only be estimated indirectly as the two lineages are morphologically indistinguishable without genetic identification. Thus, the percentage cover of each lineage was estimated by using the relative proportions of individuals from each lineage from a previous mtDNA-based population genetic study^14^.

### Genetic analyses

Genetic diversity in *P*. *perna* was assessed using data from mitochondrial DNA sequences (COI) and a suite of microsatellites loci. Only microsatellites were assessed for the invasive *M*. *galloprovincialis* since its COI diversity is very low^14^. Moreover, mitochondrial DNA evolves comparatively slowly and would only reveal historical demographic information from the species’ native European habitat^14^.

Fifty adult mussels (shell length > 4 cm) were collected from each site in November 2014, and mantle tissue weighing 20–30 mg was dissected from each mussel, preserved in 100% ethanol and stored at –20 °C. Specimens of *P*. *perna* and *M*. *galloprovincialis* were collected from edge and centre populations as shown in Fig. 1 and Table 1.

Whole genomic DNA was extracted using the CTAB method^20^. From each specimen, a piece of tissue the size of a match head was excised and placed into a labelled 1.5 ml Eppendorf tube and left to dry. One ml of CTAB buffer (2 g of cetyltrimethyl ammonium bromide powder, 1 g of polyvinyl pyrrolidone powder, 100 ml of 1 M Tris, 28 ml of 5 M NaCl, 4 ml of 0.5 M EDTA and filled up to 100 ml with deionised water) and 10 µl of Proteinase K were then added. The tubes were kept at 55 °C for three hours (or at 37 °C overnight) and occasionally shaken until tissues had dissolved completely, whereupon 500 µl of a 1:24 isoamylalcohol-chloroform mixture was added, and tubes shaken vigorously for 30 seconds. The tubes were then centrifuged at 13 000 rpm for 5 min, whereafter 500 µl of the supernatant was transferred to newly labelled 1.5 ml Eppendorf tubes. Next, 500 µl of isopropanol was added to the tubes, shaken, and the mixture was centrifuged for 10 min at 13 000 rpm. Tubes were then decanted, and DNA pellets washed twice with 700 µl of 70% ethanol, and centrifuged at 13 000 rpm for 10 minutes. All liquid was removed from the tubes, and DNA pellets were left to dry completely at room temperature for 30 min. 20 µl of dilute TE (Tris-EDTA) buffer (0.1 mM Tris-Cl [pH 7.6], 0.001 mM EDTA) was added to each Eppendorf tube, vortexed and stored in the freezer at −20 °C.

The mtDNA was amplified with the universal primers LCO1490 (5′-GGT CAA CAA ATC ATA AAG ATA TTGG -3′) and HCO2198 (5′-TAA ACT TCA GGG TGA CCA AAA AAT CA-3′)^21^. PCR reactions were performed in reaction volumes of 20 µl containing the following reagents: 2 µl of DNA template, 1.2 µl of 25 mM MgCl_2_ and 2 µl of 10x PCR buffer (Promega), 0.64 µl of 1 mM dNTP mixture (Sigma-Aldrich), 0.5 µl of forward and reverse primers (10 mM), 0.24 µl of BSA, 0.16 µl of Super-Therm *Taq* polymerase (5 units/ml, Separation Scientific SA), and 12.7 µl of distilled water. PCR amplifications were performed on a Multigene Optimax Thermal Cycler (Labnet) using the following profile: an initial denaturation step (94 °C for 2 min), 40 cycles of denaturation (94 °C for 30 sec), annealing (50 °C for 40 sec), and extension (72 °C for 1 min), and a final extension step (72 °C for 10 min). Gel electrophoresis was performed to confirm DNA amplification. PCR products were then purified with the Wizard SV Gel and PCR Clean-Up System (Promega), cycle sequenced both in the forward and reverse directions using Big Dye Terminator v3.1 Cycle Sequencing Kit (Applied Biosystems), and visualized on an ABI 3730 Genetic Analyser.

Microsatellite genotyping of *P*. *perna* and *M*. *galloprovincialis* was carried out using a set of 10 and nine loci, respectively, with markers previously reported to conform to the expectations of Linkage Disequilibrium and Hardy-Weinberg equilibrium (Table S1). A reaction volume of 18 µl of a PCR solution was used. Each tube contained the following reagents: 2 µl DNA template, 1.5 µl of 25 mM MgCl_2,_ 2 µl of 10x PCR buffer (Promega), 2 µl 1 µM dNTP mixture (Sigma-Aldrich), 0.4 µl of forward and reverse primers (10 mM), as well as 0.6 µl of universal fluorescently labelled M13 primer, 0.24 µl of BSA, 8.7 µl of deionised water, and 0.16 µl of Super-Therm *Taq* polymerase (5 units/ml, Separation Scientific SA). The PCR amplification of *P*. *perna* was performed on a GeneAmp 9700 thermocycler (PE Applied Biosystems) under the following conditions: an initial denaturation step (95 °C for 5 min), 35 cycles of denaturation (95 °C for 30 sec), annealing at a primer-specific annealing temperature (Table S1) for 30 sec and extension (72 °C for 40 sec), and a final extension step (72 °C for 20 min). For *M*. *galloprovincialis*, amplification was performed under the following conditions: an initial denaturation step (94 °C for 2 min), 30 cycles of denaturation (92 °C for 1 min), annealing at a primer-specific annealing temperature (Table S1) for 1 min and extension (72 °C for 30 sec), followed by a final extension at 72 °C for 10 min.

A post-PCR multiplexing technique was employed for both species, where loci were amplified separately and then pooled for genotyping, as previously described for *P*. *perna*^22^. The PCR products were analysed using an ABI PRISM 3730 DNA Analyser (Applied Biosystems), with GeneScan Liz 500 (Applied Biosystems) as size standard.

Mitochondrial DNA sequences of *P*. *perna* were aligned and edited in MEGA7^23^. For the microsatellite data, GENEIOUS microsatellite plug-in 1.4^24^ was used to analyse, score and bin alleles. Two researchers confirmed the scoring of alleles independently. The quality of the microsatellite data was assessed by exploring the occurrence of null alleles and by calculating departures from Linkage Disequilibrium (LD) and Hardy-Weinberg equilibrium (HWE). The proportion of null alleles per locus was calculated in FreeNA^25^, while LD and HWE were calculated in Arlequin v3.5.2.2^26^. Likelihood ratio tests for LD between all pairs of loci were calculated for each sampling location, with 1000 permutations to test for significance, two initial conditions for the expectation-maximisation algorithm, and Bonferroni correction^27^ for multiple tests. Exact tests for HWE were conducted by specifying 10^6^ steps in the Markov chain and 10^5^ dememorisation steps.

#### Genetic differentiation

The evolutionary relationships between the COI sequences of *P*. *perna* were visualized by constructing a median-joining network^27^ in PopART 1.7^28^. Samples that were not part of the species’ dominant lineage in the region where they were found were excluded from subsequent analyses (1 individual from site P2 and 1 individual from site P3); in both cases these were eastern haplotypes that were present at sites that were dominated by western haplotypes and thus reflect long-distance migration. To assess genetic differentiation within the ranges of individual lineages that may indicate reduced gene flow between centre and edge sites, population pairwise Φ_PT_ was calculated using GenAlEx 6.501^29^, and Bonferroni correction was applied to account for multiple comparisons.

STRUCTURE 2.3.4^30^ was used to assess genetic structuring in the microsatellite data of both *P*. *perna* and *M*. *galloprovincialis*, with both the admixture and ‘locprior’ models invoked to infer the ancestry of individuals. The program was run on the Lengau supercomputer at the CSIR Centre for High Performance Computing in Rondebosch, South Africa, by specifying up to five genetic clusters (*K = *1–5) with a burn-in of 1,000,000 iterations followed by 10,000,000 MCMC iterations. Each run was repeated ten times with different starting seeds to confirm that the program was run sufficiently long for the results to be consistent. Evanno’s Δ*K*^31^ was calculated with STRUCTURE HARVESTER^32^ to identify the best-supported number of distinct genetic clusters comprising each species. After confirming with CLUMPAK^33^ that all ten independent runs per *K* converged on the same solution, a barplot was created from one of the Q-matrices for *P*. *perna* with STRUCTURE PLOT 2.0^34^. Further regional genetic subdivision in *P*. *perna* was explored by analysing western and eastern sites separately, using the same settings as above. GenAlEx was used to calculate pairwise *F*_ST_ values, as well as *G”*_ST_, a genetic structure statistic similar to *F*_ST_ that is particularly suitable for microsatellites^35^. Bonferroni correction was applied as described above. The program FreeNA was used to determine whether null alleles affected pairwise *F*_ST_, with significant differences between corrected and uncorrected estimates determined by calculating 95% confidence intervals that were based on 1000 bootstrap replications.

Evolutionary relationships between the lineages were also visualized using a minimum spanning network that was obtained from the R package *Poppr* 2.3.0^36^. Average Bruvo’s distance^37^ over all loci in a population was calculated, the model for missing data was set to average addition/loss and the repeat lengths of each locus were entered.

#### Estimates of genetic diversity

For the COI data, we used Arlequin to calculate site-specific haplotype diversity *h*^38^ and nucleotide diversity π^39^. When *h* is high, this implies that a population has a large number of haplotypes, whereas a high π indicates that the haplotypes present in a population are very different from each other. Both are typical of large populations, and it was expected that *h* and π would be greater for centre sites than for edge sites. To determine whether these diversity indices differed between pairs of sites, we used the R package *genetic_diversity_diffs* 1.0.5^40^ and specified 1000 iterations to calculate Bonferroni-corrected P-values.

To assess genetic diversity for the microsatellite data, we calculated allelic richness (A_r_) in the R package *diveRsity*^41^. Compared to the more commonly used expected heterozygosity (*H*_E_), this statistic is more likely to be affected by stochastic processes in small populations at the range edge, and for that reason is more sensitive in detecting differences between centre and edge sites^9^. Significant differences in the magnitude of A_r_ between sites were identified by calculating 95% confidence intervals based on 1000 bootstrap replications, with non-overlapping confidence intervals between two sites implying significant differences. As it is not possible to calculate confidence intervals for A_r_ in *diveRsity* when rarefaction (correction for differences in sample size) is applied, we reduced the number of individuals at all sites to the number at the site with the smallest sample size by excluding individuals with the most missing data, which was 41 individuals for *P*. *perna*. No reduction was necessary for *M*. *galloprovincialis* because all sites had 48 individuals.

### Lagrangian particle simulations

To provide an independent estimate of the dispersal potential of *P*. *perna* and *M*. *galloprovincialis*, Lagrangian particle simulation was performed. The simulation followed implementations used previously^18,42,43^, and used data assembled from the Hybrid Coordinate Ocean Model (HYCOM), a product delivering ocean current fields on a daily basis (spatial resolution of 0.08°, approx. 6–9 km). This is a model forced by heat flux, precipitation, wind stress and wind speed that is able to resolve meandering currents, eddies, filaments and fronts^44^, i.e., important mesoscale oceanographic processes required to accurately simulate passively dispersing larvae^18,45^.

The region of simulation comprised ~2750 km of coastline that slightly exceeded the range from which genetic samples were obtained, from St. Helena Bay (South Africa; 32.75° S, 17.85° E) in the west to Ponta Dobela (Mozambique; 26.25° S, 32.95° E) in the east. A high-resolution polygon representing global landmasses^46^ was used to define source/sink locations 1 km apart. From these points, individual particles simulating pelagic mussel larvae were released on a daily basis, and allowed to drift at the surface of the virtual environment for up to 30 days^18^ until they eventually ended up on the shore, or were lost in the open ocean. The position of each particle was determined every hour using the bilinear interpolation of HYCOM’s ocean velocity fields. Individual trajectories were aggregated to matrices representing the probability of connectivity between every pair of coastal locations, by dividing the number of times a particle released from location *i* reached location *j*, by the total number of particles released from location *i*.

$${\rm{{\rm P}}}(i\to j)=\frac{\sum i\to j}{\sum i}$$

To account for inter-annual variability in ocean flows, the simulations were run individually for a period of 10 years (2003 to 2012), and a final asymmetrical connectivity matrix between all pairs of coastal locations was produced by averaging the annual matrices. Overall, a total of 2,697,350 particles were released.

The potential connectivity between the locations sampled for genetics was determined with direct probability estimates from the final asymmetrical matrix, and by considering stepping-stone probability estimates. For the latter approach, network analysis was implemented, with vertices being locations and edges the probabilities of asymmetrical connectivity^47^. Floyd–Warshall’s algorithm, which minimises the overall sum of log‐transformed probabilities, was used to estimate the shortest path between sampled locations, from which probabilities of connectivity were determined by a product function. Finally, clusters of locations with higher connectedness were determined by finding the optimal partitioning for maximizing the modularity index (i.e., goodness-of-fit) over all possible partitions. Dispersal simulations and network analyses were performed using the R packages *igraph*^48^, *dismo* v1.1-4^49^, *parallel* v3.6.1 (R-core), *raster* v0.46^50^, and *vegan* v2.5-6^51^.

### Ethical approval and informed consent

A research permit (RES2014/12) issued jointly by the Department of Environmental Affairs and the Department of Agriculture, Forestry and Fisheries was used to sample mussels from the South African coastline.

## Results

### Estimation of mussel cover

Mussel cover was on average 0.13% at edge sites of the western lineage, increasing to 58.47% at centre sites (Table 2). Centre sites of the eastern lineage had even greater mussel cover, on average 72.59%. Based on a neighbour-joining phylogram of mtDNA sequences^14^, the two lineages were equally represented at site P5, while at site P6, 30% of the mussels were assigned to the western and 70% to the eastern lineage. Given mussel cover of 62.2% and 52.1% at sites P5 and P6 respectively, the resulting cover for each lineage was estimated to be 33.1% (P5) and 15.6% (P6) for the western lineage, and 33.1% (P5) and 36.4% (P6) for the eastern lineage. Centre locations of *Mytilus galloprovincialis* also had higher cover than edge locations (58.3% and 0.23%, respectively).

**Table 2:** A comparison of percentage cover (%) at centre and edge sites of *Perna perna* and *Mytilus galloprovincialis*.

Species	Lineage	Code	Average % cover (SD)	Site Category
*Perna perna*	West	P1	0.14 (0.27)	edge
	West	P2	0.12 (0.22)	edge
	West	P3	57.49 (12.41)	centre
	West	P4	59.46 (13.98)	centre
	Both	P5	66.2 (26.61) − 50% West, 50% East	edge
	Both	P6	52.1 (25.7) - 30% West, 70% East	edge
	East	P7	71.65 (20.91)	centre
	East	n/a	73.53 (26.88)	centre
*Mytilus galloprovincialis*		M1	38.19 (14.67)	centre
		M2	40.22 (17.76)	centre
		M3	0.32 (0.41)	edge
		M4	0.15 (0.29)	edge

### Genetic analyses

#### Genetic structure

**Figure 2:**
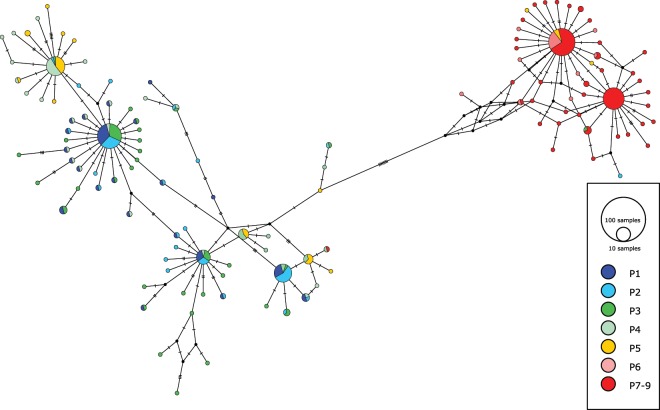
A median-joining network constructed from COI haplotypes of *Perna perna* with PopART 1.7 (http://popart.otago.ac.nz/). Haplotypes are represented by circles whose size is proportional to their frequency. Vertical lines represent the number of nucleotide differences between haplotypes, and colours represent sampling sites.

The two COI lineages of *Perna perna* were recovered as distinct genetic clusters (Fig. 2), with some evidence for migration (some typical western haplotypes were also found in the east, and eastern haplotypes were found in the west). Eastern edge individuals clustered throughout the network, but in both lineages, those individuals were limited to certain clusters.

For both lineages of *P*. *perna*, significant Φ_PT_ values were found for several pairs of sites (Tables S2 and S3), but these did not conform to the pre-defined centre and edge groupings. In the western lineage, there was a clear geographical division into western sites (edge sites P1, P2 and centre site P3) vs. eastern sites (centre site P4 and edge site P5). In the eastern lineage, the northernmost site (centre site P9) was distinct from all other sites.

FreeNA analyses indicated that null alleles were quite common for some microsatellite loci (Tables S4 and S5), although this had no significant effect on estimates of genetic structure (see next paragraph). Tests for Linkage Disequilibrium (LD) between microsatellite loci of *P*. *perna* were significant in several cases, although no pair of loci was consistently in LD at all sites (Table S6). In contrast, very few tests of *M*. *galloprovincialis* loci were significant (Table S7). Departures from Hardy-Weinberg equilibrium, which were mostly heterozygote deficits, were also much more common for *P*. *perna* (Table S8) than for *M*. *galloprovincialis* (Table S9). Such results are often found in studies on high-dispersal marine invertebrates^52^. Although the difference between the two mussel species is difficult to explain, it may be related to complex small-scale selective forces in *P*. *perna* that result in skewed sex ratios, reducing the chances of random mating^53^ and potentially producing localized Wahlund effects^54^ where migrants from adjacent but different habitats have reduced fitness^55^. As analyses of microsatellite data produced results that were very similar to those from the mtDNA data, and null alleles did not significantly affect genetic structure (see next paragraph), we decided not to exclude any loci from subsequent analyses.

*F*_ST_ and *G”*_ST_ values (Table S10) were significantly different between most pairs of sites even after Bonferroni correction. The only pairs of sites showing non-significant differences were found in the same region (west or east), but no clear trend was evident between centre and edge sites within a particular region. A comparison of *F*_ST_ values corrected for null alleles with the uncorrected *F*_ST_ values showed that these were not significantly different, as 95% confidence intervals overlapped in all cases (Fig. S1).

**Figure 3:**
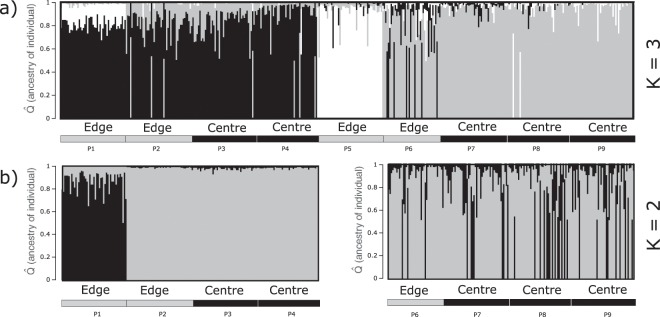
Barplots of *Perna perna* microsatellite data constructed using STRUCTURE 2.3.4. Each vertical bar represents a single individual, and colours indicate the association of these with genetic clusters; (**a**) result for all nine sites (P1-P9) and three clusters; (**b**) results for western and eastern sites separately for two clusters, excluding site P5. Association of individuals with centre and edge sites is shown below the barplots. The figures were constructed in STRUCTURE PLOT 2.0 (http://omicsspeaks.com/strplot2/) and modified in InkScape 0.92.4 (https://inkscape.org).

STRUCTURE analyses revealed that the best number of distinct clusters based on Evanno’s Δ*K* was not 2 (western and eastern lineage) but 3 (Fig. S2), with most individuals from site P5 being part of a distinct cluster (Fig. 3a). STRUCTURE analyses using only western sites (P1–P4) or eastern sites (P6–P9) further revealed that the westernmost edge site (P1) was distinct from other western sites (P2-P4), while no further subdivision was evident in the east (Fig. 3b).

The minimum-spanning network for the microsatellite data of *P*. *perna* (Fig. S3) did not recover distinct western and eastern evolutionary lineages, although trends are evident (e.g., the cluster on the left mostly comprises eastern sites, and that on the top right has particularly large numbers of western sites).

Evanno’s Δ*K* method supported two genetic clusters of *M*. *galloprovincialis* (Fig. S4). However, this seems to be a result of the method being unable to provide a result for *K* = 1. A STRUCTURE barplot did not identify any spatial genetic structure but assigned each individual to both clusters (Fig. S4), and there is thus no evidence for more than one genetic cluster. This conclusion is supported by the minimum-spanning network for this species, which showed no spatial trends (Fig. S5), and by the fact that, with a single exception, *F*_ST_ and *G”*_ST_ values were not significantly different for all pairs of sites (Table S11). Again, *F*_ST_ values corrected for null alleles were not significantly different from uncorrected values (Fig. S1).

#### Comparisons of genetic diversity indices

On the basis of the genetic structure results for *P*. *perna*, several adjustments to the *a priori* assignment of sites to either centre or edge populations shown in Fig. 1 were considered necessary. First, site P5 could not be clearly assigned to either western or eastern lineages, and for that reason, diversity indices were either reported separately for the individuals associated with each lineage (mtDNA data), or it was treated as a distinct site (microsatellite data). Second, site P6, which was previously identified as being located in a contact area of the two lineages^14^ (Fig. 1, Table 2), an arrangement that was also supported by the Lagrangian modelling results (see below), had only eastern COI haplotypes in the present study. It was also not clearly distinct from eastern sites based on the microsatellite data (Fig. 3b), and for that reason was treated as an edge population of the eastern lineage.

**Figure 4:**
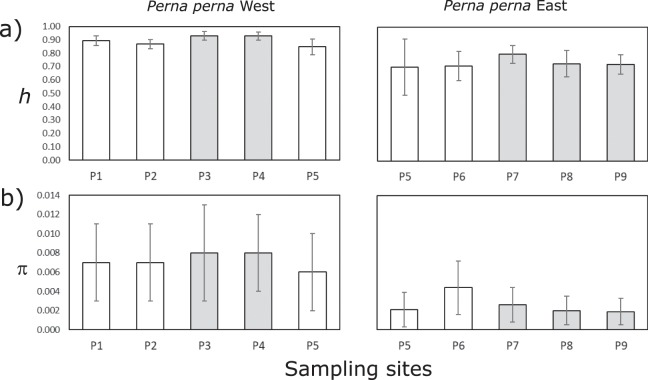
Comparisons of COI-based genetic diversity indices between centre (grey bars) and edge (white bars) sites of the western and eastern lineages of the brown mussel, *Perna perna*: a) haplotype diversity *h* and b) nucleotide diversity π. Values are means and error bars are standard deviations. Please see Tables S6 and S7 for details.

For the western lineage of *P*. *perna*, significant differences in haplotype diversity (*h*) were found in three cases (Table S12), but these were no longer significant after Bonferroni correction and thus likely false positives (Fig. 4a). Nucleotide diversity (π) was mostly lower for the eastern lineage than for the western lineage, but no significant differences between centre and edge sites of the same lineage were identified (Fig. 4b, Table S13).

**Figure 5:**
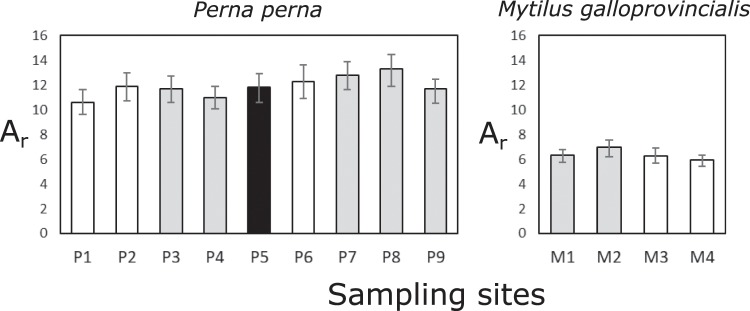
Comparisons of microsatellite-based allelic richness (A_r_) between centre (grey bars) and edge (white bars) sites of the western and eastern lineages of the brown mussel, *Perna perna* and the invasive *Mytilus galloprovincialis*. Error bars are 95% confidence intervals, and overlap between these indicates that genetic diversity indices are not significantly different. In this case, individuals of *P*. *perna* could not always be readily assigned to either the western or the eastern lineage, and site P5 (which was distinct from western and eastern sites, Fig. 3a) is shown in black.

Allelic richness (A_r_) based on the microsatellite data is reported together for the two lineages of *P*. *perna* because they were not as clearly differentiated as was the case for the mtDNA data (Figs. 3 and S3). This statistic was distinctly larger for the native *P*. *perna* than for the invasive *M*. *galloprovincialis* (Fig. 5). In this case, there were no clear differences in genetic diversity between the two lineages of *P*. *perna*. Ninety-five percent confidence intervals overlapped for all the centre and edge sites of both species, which implies no significant differences.

### Lagrangian simulations

**Figure 6:**
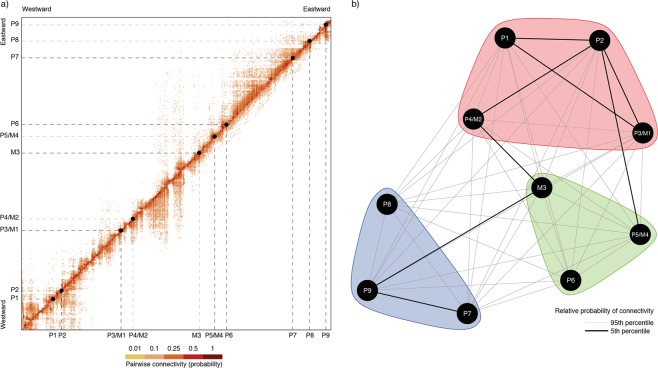
Pairwise connectivity inferred with Lagrangian particle simulations; (**a**) connectivity matrix between all coastal locations in South Africa averaged for a 10-year period; (**b**) connectivity and clustering between locations from which genetic samples were collected (considering the stepping-stone scenario) created in InkScape 0.92.4 (https://inkscape.org). Thicker lines indicate higher probabilities of connectivity (please refer to Tables S15 and S16).

The particle simulations over the 10-year period showed a sharp decline of potential connectivity with distance (Fig. S6), with high probabilities for larval retention near the parent habitat (matrix diagonals in Fig. 6a) and mean traveled distances of 59.35 ± 69.86 km in 4.97 ± 5.21 days (maximum 1002.81 km; Table S14; Mov S1). When considering direct connectivity, only two pairs of locations sampled for genetics were potentially connected by ocean currents (P2 to P1 and P4 to P3; Fig. 6a, Table S15), but when considering a stepping-stone scenario, all pairs were potentially connected, albeit with skewed probabilities with increasing distance (Table S16). This scenario recovered three main clusters with higher connectedness that, overall, conformed well to the regional clusters identified using genetic methods: a western cluster including locations P1 to P4, as well as M1 and M2; a central cluster including locations P5, P6, M3 and M4; and an eastern cluster including locations P7 to P9 (Fig. 6b). The main differences compared to the genetic results are thus a) the distinctness of the originally defined eastern edge sites (P5 and P6) from eastern centre sites (P7–P9) in *P*. *perna* and b) a clear division into centre (M1 and M2) and edge (M3 and M4) sites in *M*. *galloprovincialis*.

## Discussion

Originally developed in the context of population genetics^56^, the Abundant-Centre Hypothesis (ACH) has subsequently been extended to include ecological aspects of species, such as physiological performance^57^. Empirical studies do not always support this hypothesis, and it is useful to consider the definition of an edge population. Two definitions have been suggested^56^: populations whose size fluctuates dramatically and which have a high likelihood of becoming extinct, and populations whose members are sparse and physiologically stressed. Such stress could be due to environmental conditions or competitive interactions^58^. This makes the important point that marginal populations are not necessarily found at the periphery of a species’ distribution^59^, and this in turn is compatible with the observation that species’ ranges need not contract from the periphery to the centre^60^. In terms of testing the ACH, coastlines have the advantage of being essentially one-dimensional, making it relatively easy to identify central and peripheral populations. The South African coastline, apart from being much more linear than many other coastlines, has the additional advantage that it encompasses a wide range of climatic and oceanographic conditions that define at least four marine biogeographic provinces^12^. These are separated by biogeographical transition zones that represent the range edge in numerous species^61^ and that are so distinct environmentally that they can drive the evolution of genetic lineages endemic to these transition zones^62,63^. On this basis, we have made the assumption that populations near the species’ distributional limits are indeed marginal^56^. The clear spatial arrangement of centre and edge regions theoretically offers an ideal setting that facilitates multiple tests of the ACH. The rejection of the hypothesis in two evolutionary lineages of the native mussel *Perna perna* and in the invasive *Mytilus galloprovincialis* provides insights into the conditions that prevent species from developing the genetic patterns expected to result from the ACH.

Genetic structure results for mtDNA and microsatellite data of *P*. *perna* were similar in that both markers identified the western and eastern lineages. However, smaller-scale genetic structure did not clearly conform to the pre-defined divisions into center and edge populations, and also differed between markers. The western COI lineage could be further subdivided into a western and an eastern group (with a boundary between sites P3 and P4) whose ranges did not match the biogeography-based division into centre and edge sites, but which may nonetheless reflect a thermal gradient in this area^64,65^. A similar result was found for the microsatellite data, although the location of the boundary differed (it was located between sites P1 and P2) and may reflect marker-specific differences in lineage sorting^66^ or selection^67^. Similarly, the distinctness of the COI haplotypes from the northernmost site (P9), which was not found with microsatellites, matches the location of previously reported phylogeographic breaks that separate subtropical from tropical fauna^61,68–70^, and may thus also reflect differential adaptation across a temperature gradient.

In terms of genetic diversity, there were clear trends between species or lineages, but none were related to differences between centre and edge sites. Genetic diversity indices were higher in western *P*. *perna* than in eastern *P*. *perna* (mtDNA data), and higher in the microsatellite data of *P*. *perna* than in those of *M*. *galloprovincialis*. As mentioned earlier, no mtDNA data were generated for the latter species because its genetic diversity is very low^14^. In addition, given the slow mutation rate of this marker, its genetic diversity likely still reflects the species’ demographic history in the northern hemisphere prior to its introduction to southern Africa. However, the microsatellite data were also considerably less variable than in *P*. *perna*, possibly because of a reduction in diversity during the founder event(s) that occurred at the beginning of the species’ invasion of southern Africa in the 1970s.

A reduction in genetic diversity towards the range edge expected under the ACH is predicted on the basis of a combination of smaller population size and increased spatial isolation from the centre of the range^71^. Hence, even when the abundance of mussels is lower in edge populations, reduced genetic diversity is only expected when migration from the centre is so low that some alleles are so rare in the periphery that they are not represented in a reasonably large genetic sample.

A recent study on two species of the gastropod genus *Crepidula* with very different dispersal potential identified support for the ACH for both species^72^. Although the absolute reduction in genetic diversity that was found for marginal populations was relatively small, this implies that dispersal capability is irrelevant. In our case, all three taxa have relatively long-lived planktonic larvae and are expected to have a high capacity for dispersal, although empirical evidence suggests that the numbers of larvae undergoing long-range dispersal on this coast may not be high in terms of ecological connectivity^73^. Accordingly, previous research using mtDNA sequence data of *P*. *perna* identified surprisingly well-developed genetic structure within the western lineage that was driven by coastal topography^55^ and even resulted in sex-biased genetic structure between bays and the open coast^53^. In the present study, we identified numerous examples of genetic structure that imply some degree of spatial isolation, but the data from neither genetic marker supported division into groups of centre or edge sites.

The Lagrangian modelling allowed the assignment of locations to three distinct clusters with high within-group and lower between-group connectedness, the clusters largely coinciding with the biogeography-linked regional patterns of genetic structure found for *P*. *perna*. Interestingly, the modelling results supported the distinctness of the originally-defined edge populations of both species, even though their distinctness was not strongly supported by the present genetic data. While a 30-day dispersal period may potentially generate connectivity over a distance of up to 1000 km, connectivity events on average were in the order of only ~60 km in ~5 days^43^. Nevertheless, the possibility of year-to-year stepping-stone connectivity^47^, together with the mussels’ high reproductive output and long pelagic larval phases, may facilitate the spread of genes across each species’ entire range.

The rejection of the genetic expectations of the ACH, despite: (a) lower population densities of edge populations, (b) clear departures from the expectations of panmixia in *P*. *perna*, and (c) reduced larval connectivity between some of the originally-defined centre and edge populations identified using Lagrangian modelling, implies that the genetic consequences of the ACH may only become evident when levels of connectivity are so low that they manifest at the evolutionary (rather than ecological) timescale. In addition, population sizes even of edge populations may be sufficiently large to prevent diversity-reducing factors that affect small populations (e.g. genetic bottlenecks). Hence, occasional larval exchange between large central and peripheral populations of South African marine mussels may prevent geographic genetic divergence, and be sufficient to nullify the conditions expected to underpin an ACH effect. We suggest that the genetic predictions of the ACH are unlikely to be supported by species with high dispersal capabilities such as those with planktonic, wind- or vector-driven dispersal of propagules.

## Supplementary information

Supplementary information.

## Data Availability

DNA sequences have been submitted to GenBank (accession numbers MN720954–MN721291). Microsatellite data and an animation of larval dispersal are available from figshare (10.6084/m9.figshare.8910812).
