# Supplementary material for: Rejection of the genetic implications of the “Abundant Centre Hypothesis” in marine mussels
Source: Sci Rep. 2020 Jan 17;10:604. doi: 10.1038/s41598-020-57474-0 (PMC6969206; doi:10.1038/s41598-020-57474-0)
Supplement: Supplementary file 1 — Supplementary information. [file 41598_2020_57474_MOESM1_ESM.docx]

**Rejection of the genetic implications of the “Abundant Centre Hypothesis” in marine mussels**

**Noxolo N. Ntuli^1^, Katy R. Nicastro^2^, Gerardo I. Zardi^3^, Jorge Assis^2^, Christopher D. McQuaid^3^ & Peter R. Teske^1*^**

^1^Centre for Ecological Genomics and Wildlife Conservation, Department of Zoology, University of Johannesburg, Auckland Park 2092, South Africa; ^2^CCMAR - Centro de Ciências do Mar, Universidade do Algarve, Faro, Portugal; ^3^Department of Zoology and Entomology, Rhodes University, Grahamstown 6140, South Africa

*Corresponding author, email: [pteske101@gmail.com](mailto:pteske101@gmail.com)

**Fig. S1** Comparisons between *F*_ST_ values between all pairs of sites for *Perna perna* (top) and *Mytilus galloprovicialis* (bottom). Grey bars represent uncorrected *F*_ST_ values and white bars are *F*_ST_ corrected for the presence of null alleles in FreeNA. Error bars are 95% confidence intervals. *F*_ST_ values are not significantly different when these overlap.


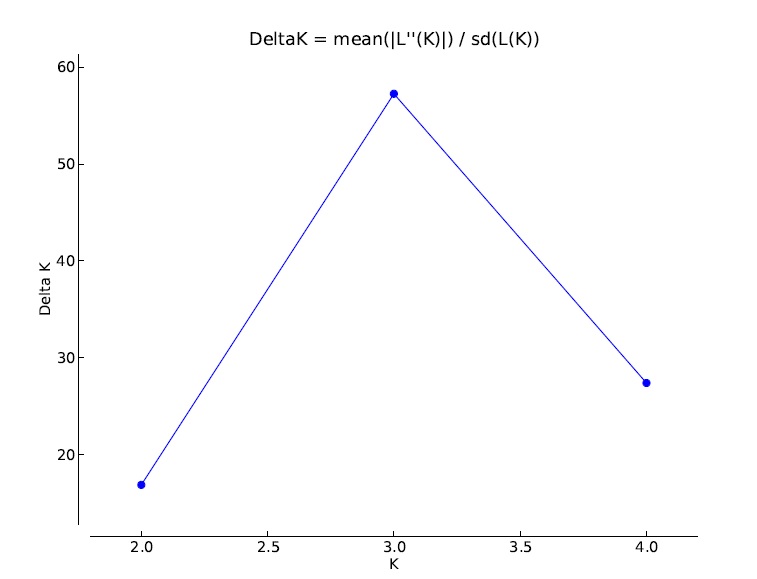


**Fig. S2** Evanno’s Δ*K* for 367 individuals of eight *Perna perna* populations. The inferred number of genetic clusters (*K*) is three.


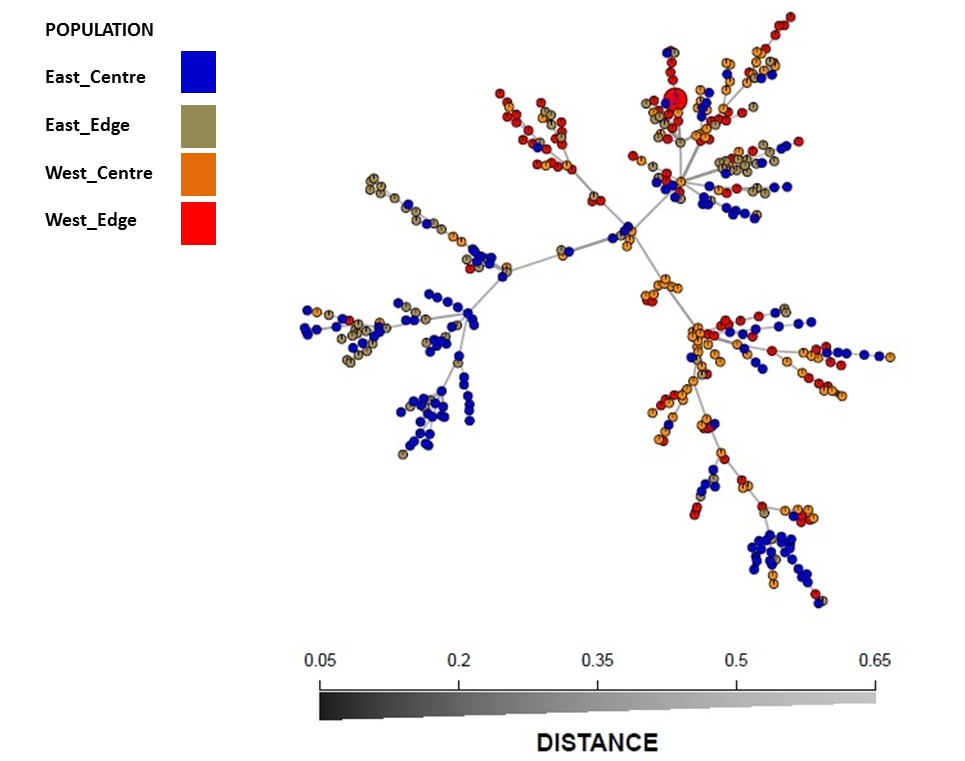


**Fig. S3** Microsatellite-based network for *Perna perna* populations constructed in the R package *Poppr* 2.3.0 (https://cran.r-project.org/web/packages/poppr/). Haplotypes are represented by circles and the size of each is proportional to the haplotype frequency. The smallest circles represent single individuals. The line between each haplotype represents mutational changes and the shortest connections indicate one mutational step.

**
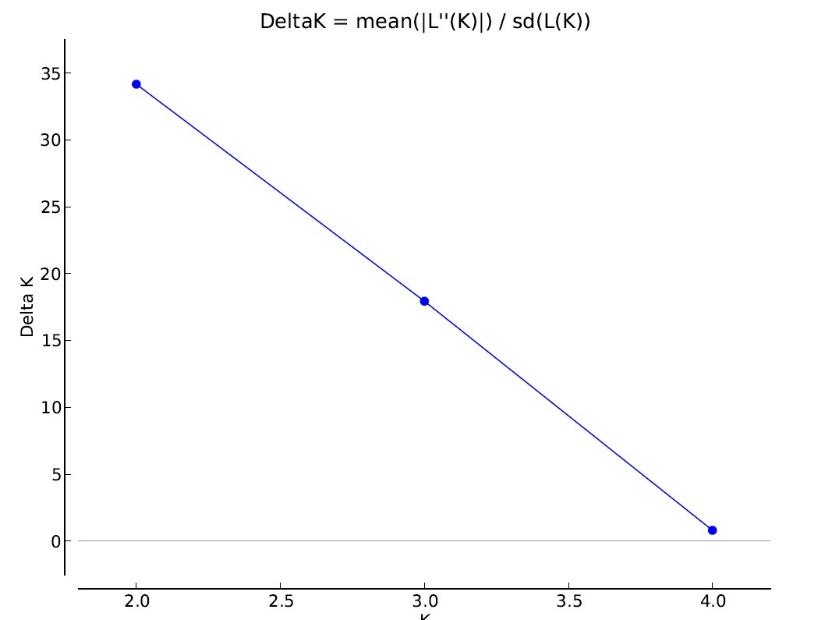
**

**
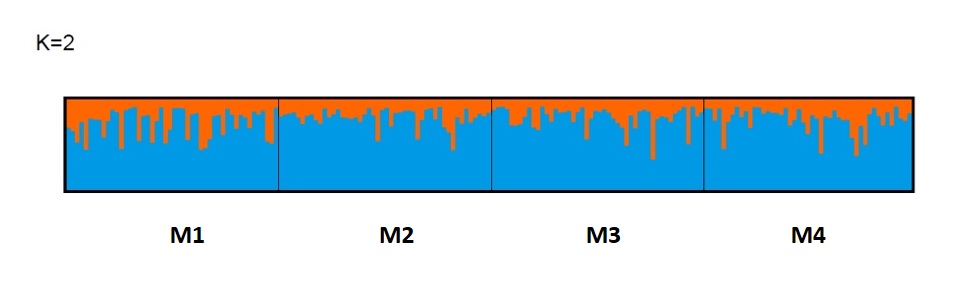
Fig. S4** Top: Evanno’s Δ*K* for 191 individuals of *Mytilus galloprovincialis* collected at four sites. The assumed number of genetic clusters (*K*) is two; bottom: barplot of *Mytilus galloprovincialis* microsatellite data based on analyses performed in STRUCTURE 2.3.4, assuming *K*=2 based on Evanno’s Δ*K*. Each vertical bar represents a single individual, and colours indicate the inferred association of these with the inferred two genetic clusters.

*
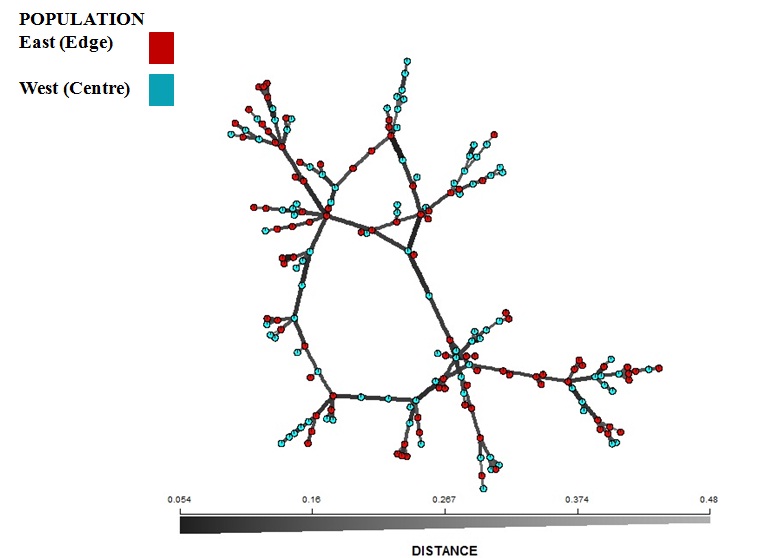
*

**Fig. S5** Microsatellite-based minimum-spanning network for *Mytilus galloprovincialis* populations constructed in the R package *Poppr* 2.3.0 (https://cran.r-project.org/web/packages/poppr/). Haplotypes are represented by circles and the size of each is proportional to the haplotype frequency. The smaller haplotypes represent single individuals. The line between each haplotype represents mutational changes and the shortest connections indicate one mutational step.

**Fig. S6** Relationship between marine distances and probabilities of connectivity between all pairs of cells inferred with the simulations running in South Africa.

**Table S1** Characterization the microsatellite loci for *Perna perna* and *Mytilus galloprovincialis*.

| Species | Locus | Primer sequences | Repeat motif | Annealing temperature (˚C) |
| --- | --- | --- | --- | --- |
| *Perna perna* | P01 | **F**-TGGAACTTAGGGCCTTC  **R**-TCCAATTCTGTGAAATC | (AC)_13_ | 56 |
|  | P02 | **F**-CCCGGTTTTAAGTGGTA  **R**-AGCAAACGAAGGACAA | (GT)_15_ | 57 |
|  | P05 | **F**-TCAGTGCCGCGTGATA  **R**-TCCAATTACGTTTGTTT | (TG)_15_ | 56 |
|  | P08 | **F-**AATGTTCAATGTCGACA  **R**-TTCTGAAGCACTGGAT | (ACAG)_7_ | 59 |
|  | P11 | **F**-TTAACGTTGAAATCCGT  **R**-CATTCCAATCCCACGCA | (GT)_9_ | 58 |
|  | P16 | **F**-TGTGATTTAAAGTTGGA  **R**-TGATTGGATCAAAATT | (AC)_26_ | 56 |
|  | P20 | **F**-ATGTCAATGTGCACAAC  **R**-CGTGTATTGGCGACTTT | (ACAG)_15_ | 55 |
|  | P26 | **F**-CACCACCCTTACAAAGA  **R**-TTTCACTTGGCGATTAG | (AC)_11_ | 53 |
|  | P27 | **F**-CCCAATTTAACGGGAA  **R**-TTATCATCACCACTTTA | (AC)_27_ | 56 |
|  | P29 | **F**-TTCCATTTCTAGACATC  **R**-TCAGGTGACAGCAGCC | (AC)_11_ | 56 |
|  |  |  |  |  |
| *Mytilus galloprovincialis* | Mg3^1^ | **F**-AAACTAAAAACTTCATCTA  **R**-AAGCAATCCAAAGTGAGA | (TG)_5_ | 60 |
|  | Mg181^2^ | **F**-CTGCTTCAGGTTTTATGTC  **R**-TCTGACAAATTGGCTTTTA | (CGTC)_5_ | 56 |
|  | MGE001^3^ | **F**-TGATAAGTAGCAGGTGGC  **R**-TAAGGATTGTGGGTAAGAA | (TA)_7_ | 59 |
|  | MGE005^3^ | **F**- CGTTGCCATCGTTTATTTT  **R**- GTTGTAAGTCGTTTATTTT | (TGA)_6_ | 59 |
|  | MGE007^3^ | **F**- TTGAGGGAAAGGGTATGA  **R**-GAGGATTAGATGACTGAGATG | (CAA)_5_ | 59 |
|  | MGE008^3^ | **F**- TGCTAAAAGTAATAAGACAGAT  **R**- GAGACCTCCAATAAATAAAA | (AT)_8_ | 59 |
|  | USC22^4^ | **F**-CCACAGGCACAACAAGTG  **R**-GCCACGTCTATAATGGCA | (TGTA)_4_ | 58 |
|  | USC31^4^ | **F**-TTCATCGTCTTCGCAGCTT  **R**-CATTTACCTGTACCTTGCA | (CAA)10 | 55 |
|  | USC42^4^ | **F**-CTACCGGGCCTCATTTATC  **R**-GCATCGTATTACCGGAGC | (ATTT)4 | 59 |

All primers for *P. perna* were developed by Coelho et al. 2012. Superscript numbers indicate primers used for *M. galloprovincialis*: ^1^Presa et al. 2002, ^2^Varela et al. 2007, ^3^Yu and Li 2007, ^4^Vera et al. 2010.

| **Table S2** Pairwise Φ_PT_ for COI sequences of the western lineage of *Perna perna;* below diagonal: Φ_PT_; above diagonal: P-value. These were adjusted for multiple comparisons using Bonferroni correction: *corrected P-value for 5 sites at α=0.05: 0.01; **corrected P-value for 5 sites at α=0.01: 0.002. Grey shading: pairwise comparisons of Centre vs. Edge sites (Edge West or Edge East). | | | | | | | |
| --- | --- | --- | --- | --- | --- | --- | --- |
|  |  |  | Edge West | | Centre | | Edge East |
| a) | Category | Site | P1 | P2 | P3 | P4 | P5 |
|  | Edge West | P1 | -- | 0.104 | 0.455 | 0.001** | 0.000** |
|  |  | P2 | 0.020 | -- | 0.032 | 0.000** | 0.000** |
|  | Centre | P3 | 0.000 | 0.034 | -- | 0.000** | 0.000** |
|  |  | P4 | 0.089 | 0.113 | 0.094 | -- | 0.268 |
|  | Edge East | P5 | 0.190 | 0.194 | 0.186 | 0.006 | -- |

| **Table S3** Pairwise Φ_PT_ for COI sequences of the eastern lineage of *Perna perna;* below diagonal: Φ_PT_; above diagonal: P-value. These were adjusted for multiple comparisons using Bonferroni correction: **corrected P-value for 5 sites at α=0.01: 0.002. Grey shading: pairwise comparisons of Centre vs. Edge sites. | | | | | | | |
| --- | --- | --- | --- | --- | --- | --- | --- |
|  |  |  | Edge | | Centre | | |
| a) | Category | Site | P5 | P6 | P7 | P8 | P9 |
|  | Edge | P5 | -- | 0.468 | 0.295 | 0.360 | 0.000** |
|  |  | P6 | 0.000 | -- | 0.025 | 0.014 | 0.000** |
|  | Centre | P7 | 0.022 | 0.032 | -- | 0.847 | 0.000** |
|  |  | P8 | 0.022 | 0.023 | 0.000 | -- | 0.000** |
|  |  | P9 | 0.413 | 0.339 | 0.234 | 0.305 | -- |

| **Table S4.** Frequency of null alleles in the microsatellite data of *Perna perna* calculated using FreeNA. | | |
| --- | --- | --- |
| Locus No. | Population No. | Frequency of null alleles |
| 1 | 1 | 0.02673 |
| 1 | 2 | 0.00095 |
| 1 | 3 | 0.06798 |
| 1 | 4 | 0.05912 |
| 1 | 5 | 0.19550 |
| 1 | 6 | 0.16390 |
| 1 | 7 | 0.10546 |
| 1 | 8 | 0.09721 |
| 1 | 9 | 0.14883 |
| 2 | 1 | 0.22878 |
| 2 | 2 | 0.03871 |
| 2 | 3 | 0.00000 |
| 2 | 4 | 0.00000 |
| 2 | 5 | 0.00309 |
| 2 | 6 | 0.01594 |
| 2 | 7 | 0.09922 |
| 2 | 8 | 0.00787 |
| 2 | 9 | 0.05923 |
| 3 | 1 | 0.00012 |
| 3 | 2 | 0.12570 |
| 3 | 3 | 0.00000 |
| 3 | 4 | 0.05716 |
| 3 | 5 | 0.04597 |
| 3 | 6 | 0.10627 |
| 3 | 7 | 0.07919 |
| 3 | 8 | 0.07250 |
| 3 | 9 | 0.04456 |
| 4 | 1 | 0.14431 |
| 4 | 2 | 0.17470 |
| 4 | 3 | 0.19051 |
| 4 | 4 | 0.11752 |
| 4 | 5 | 0.05583 |
| 4 | 6 | 0.01036 |
| 4 | 7 | 0.04103 |
| 4 | 8 | 0.03854 |
| 4 | 9 | 0.00008 |
| 5 | 1 | 0.29270 |
| 5 | 2 | 0.27710 |
| 5 | 3 | 0.29051 |
| 5 | 4 | 0.21847 |
| 5 | 5 | 0.34537 |
| 5 | 6 | 0.39875 |
| 5 | 7 | 0.38615 |
| 5 | 8 | 0.32203 |
| 5 | 9 | 0.27351 |
| 6 | 1 | 0.37355 |
| 6 | 2 | 0.28284 |
| 6 | 3 | 0.17663 |
| 6 | 4 | 0.23703 |
| 6 | 5 | 0.34708 |
| 6 | 6 | 0.29884 |
| 6 | 7 | 0.25110 |
| 6 | 8 | 0.28408 |
| 6 | 9 | 0.37567 |
| 7 | 1 | 0.12291 |
| 7 | 2 | 0.27359 |
| 7 | 3 | 0.22753 |
| 7 | 4 | 0.36413 |
| 7 | 5 | 0.15714 |
| 7 | 6 | 0.15985 |
| 7 | 7 | 0.07461 |
| 7 | 8 | 0.16351 |
| 7 | 9 | 0.21587 |
| 8 | 1 | 0.11009 |
| 8 | 2 | 0.24605 |
| 8 | 3 | 0.25056 |
| 8 | 4 | 0.29086 |
| 8 | 5 | 0.25421 |
| 8 | 6 | 0.22113 |
| 8 | 7 | 0.26510 |
| 8 | 8 | 0.25696 |
| 8 | 9 | 0.24931 |
| 9 | 1 | 0.30413 |
| 9 | 2 | 0.41717 |
| 9 | 3 | 0.33677 |
| 9 | 4 | 0.23365 |
| 9 | 5 | 0.24854 |
| 9 | 6 | 0.32413 |
| 9 | 7 | 0.37083 |
| 9 | 8 | 0.34918 |
| 9 | 9 | 0.39045 |
| 10 | 1 | 0.19183 |
| 10 | 2 | 0.13805 |
| 10 | 3 | 0.10703 |
| 10 | 4 | 0.10776 |
| 10 | 5 | 0.22777 |
| 10 | 6 | 0.35604 |
| 10 | 7 | 0.40328 |
| 10 | 8 | 0.35979 |
| 10 | 9 | 0.37345 |

| **Table S5.** Frequency of null alleles in the microsatellite data of *Mytilus galloprovincialis* calculated using FreeNA. | | |
| --- | --- | --- |
| Locus No. | Population No. | Frequency of null alleles |
| 1 | 1 | 0.06849 |
| 1 | 2 | 0.00002 |
| 1 | 3 | 0.03702 |
| 1 | 4 | 0.10402 |
| 2 | 1 | 0.00000 |
| 2 | 2 | 0.01308 |
| 2 | 3 | 0.00927 |
| 2 | 4 | 0.01158 |
| 3 | 1 | 0.11466 |
| 3 | 2 | 0.19879 |
| 3 | 3 | 0.21425 |
| 3 | 4 | 0.24271 |
| 4 | 1 | 0.11410 |
| 4 | 2 | 0.09929 |
| 4 | 3 | 0.07292 |
| 4 | 4 | 0.11469 |
| 5 | 1 | 0.06405 |
| 5 | 2 | 0.03690 |
| 5 | 3 | 0.05467 |
| 5 | 4 | 0.04879 |
| 6 | 1 | 0.05230 |
| 6 | 2 | 0.06618 |
| 6 | 3 | 0.01962 |
| 6 | 4 | 0.08418 |
| 7 | 1 | 0.00001 |
| 7 | 2 | 0.00001 |
| 7 | 3 | 0.00000 |
| 7 | 4 | 0.00003 |
| 8 | 1 | 0.09344 |
| 8 | 2 | 0.11556 |
| 8 | 3 | 0.09388 |
| 8 | 4 | 0.18801 |
| 9 | 1 | 0.01353 |
| 9 | 2 | 0.09623 |
| 9 | 3 | 0.07003 |
| 9 | 4 | 0.14470 |

| **Table S6** Tests for linkage disequilibrium for 10 *Perna perna* microsatellites at each of 9 sites. Significance is indicated by a 1, non-significance by a 0. | | | | | | | | | | |
| --- | --- | --- | --- | --- | --- | --- | --- | --- | --- | --- |
|  |  | Locus no. | | | | | | | | |
| Site |  | 1 | 2 | 3 | 4 | 5 | 6 | 7 | 8 | 9 |
| P1 | 2\| | 0 |  |  |  |  |  |  |  |  |
|  | 3\| | 0 | 0 |  |  |  |  |  |  |  |
|  | 4\| | 0 | 1 | 0 |  |  |  |  |  |  |
|  | 5\| | 0 | 0 | 0 | 0 |  |  |  |  |  |
|  | 6\| | 0 | 1 | 0 | 1 | 1 |  |  |  |  |
|  | 7\| | 0 | 0 | 0 | 0 | 0 | 1 |  |  |  |
|  | 8\| | 0 | 0 | 0 | 0 | 0 | 0 | 1 |  |  |
|  | 9\| | 0 | 0 | 0 | 0 | 1 | 1 | 1 | 0 |  |
|  | 10\| | 0 | 0 | 0 | 1 | 1 | 0 | 0 | 0 | 0 |
|  |  |  |  |  |  |  |  |  |  |  |
| P2 | 2\| | 0 |  |  |  |  |  |  |  |  |
|  | 3\| | 0 | 0 |  |  |  |  |  |  |  |
|  | 4\| | 0 | 0 | 0 |  |  |  |  |  |  |
|  | 5\| | 0 | 0 | 1 | 0 |  |  |  |  |  |
|  | 6\| | 0 | 0 | 0 | 0 | 1 |  |  |  |  |
|  | 7\| | 0 | 0 | 0 | 1 | 1 | 1 |  |  |  |
|  | 8\| | 0 | 0 | 0 | 0 | 1 | 1 | 0 |  |  |
|  | 9\| | 0 | 0 | 0 | 0 | 1 | 1 | 1 | 1 |  |
|  | 10\| | 0 | 0 | 0 | 0 | 1 | 0 | 0 | 0 | 1 |
|  |  |  |  |  |  |  |  |  |  |  |
| P3 | 2\| | 0 |  |  |  |  |  |  |  |  |
|  | 3\| | 0 | 0 |  |  |  |  |  |  |  |
|  | 4\| | 0 | 0 | 0 |  |  |  |  |  |  |
|  | 5\| | 0 | 0 | 0 | 0 |  |  |  |  |  |
|  | 6\| | 0 | 0 | 0 | 1 | 1 |  |  |  |  |
|  | 7\| | 0 | 0 | 0 | 0 | 0 | 1 |  |  |  |
|  | 8\| | 0 | 0 | 0 | 1 | 0 | 1 | 1 |  |  |
|  | 9\| | 1 | 0 | 0 | 1 | 1 | 1 | 1 | 1 |  |
|  | 10\| | 0 | 0 | 0 | 0 | 0 | 0 | 0 | 0 | 0 |
|  |  |  |  |  |  |  |  |  |  |  |
| P4 | 2\| | 0 |  |  |  |  |  |  |  |  |
|  | 3\| | 0 | 1 |  |  |  |  |  |  |  |
|  | 4\| | 0 | 0 | 0 |  |  |  |  |  |  |
|  | 5\| | 1 | 0 | 1 | 0 |  |  |  |  |  |
|  | 6\| | 0 | 0 | 0 | 0 | 1 |  |  |  |  |
|  | 7\| | 0 | 1 | 0 | 1 | 1 | 1 |  |  |  |
|  | 8\| | 0 | 0 | 0 | 1 | 0 | 0 | 1 |  |  |
|  | 9\| | 0 | 0 | 1 | 0 | 1 | 1 | 1 | 1 |  |
|  | 10\| | 0 | 0 | 0 | 0 | 0 | 0 | 0 | 0 | 0 |
|  |  |  |  |  |  |  |  |  |  |  |
| P5 | 2\| | 0 |  |  |  |  |  |  |  |  |
|  | 3\| | 0 | 0 |  |  |  |  |  |  |  |
|  | 4\| | 0 | 0 | 0 |  |  |  |  |  |  |
|  | 5\| | 1 | 0 | 0 | 0 |  |  |  |  |  |
|  | 6\| | 0 | 0 | 0 | 0 | 0 |  |  |  |  |
|  | 7\| | 0 | 0 | 0 | 0 | 1 | 1 |  |  |  |
|  | 8\| | 0 | 0 | 0 | 0 | 0 | 1 | 0 |  |  |
|  | 9\| | 0 | 0 | 0 | 0 | 0 | 1 | 1 | 0 |  |
|  | 10\| | 0 | 0 | 0 | 0 | 0 | 0 | 0 | 0 | 0 |
|  |  |  |  |  |  |  |  |  |  |  |
| P6 | 2\| | 0 |  |  |  |  |  |  |  |  |
|  | 3\| | 0 | 0 |  |  |  |  |  |  |  |
|  | 4\| | 0 | 0 | 0 |  |  |  |  |  |  |
|  | 5\| | 1 | 0 | 1 | 1 |  |  |  |  |  |
|  | 6\| | 0 | 0 | 0 | 0 | 0 |  |  |  |  |
|  | 7\| | 0 | 0 | 0 | 0 | 1 | 1 |  |  |  |
|  | 8\| | 0 | 0 | 0 | 0 | 0 | 0 | 0 |  |  |
|  | 9\| | 1 | 0 | 0 | 0 | 1 | 1 | 1 | 1 |  |
|  | 10\| | 0 | 0 | 0 | 0 | 1 | 1 | 1 | 0 | 1 |
|  |  |  |  |  |  |  |  |  |  |  |
| P7 | 2\| | 0 |  |  |  |  |  |  |  |  |
|  | 3\| | 0 | 0 |  |  |  |  |  |  |  |
|  | 4\| | 0 | 0 | 0 |  |  |  |  |  |  |
|  | 5\| | 0 | 0 | 1 | 0 |  |  |  |  |  |
|  | 6\| | 0 | 0 | 0 | 0 | 1 |  |  |  |  |
|  | 7\| | 0 | 1 | 0 | 0 | 0 | 1 |  |  |  |
|  | 8\| | 0 | 0 | 0 | 0 | 1 | 1 | 0 |  |  |
|  | 9\| | 0 | 1 | 0 | 0 | 1 | 1 | 1 | 1 |  |
|  | 10\| | 0 | 0 | 0 | 0 | 1 | 0 | 0 | 1 | 1 |
|  |  |  |  |  |  |  |  |  |  |  |
| P8 | 2\| | 0 |  |  |  |  |  |  |  |  |
|  | 3\| | 0 | 0 |  |  |  |  |  |  |  |
|  | 4\| | 0 | 0 | 0 |  |  |  |  |  |  |
|  | 5\| | 0 | 0 | 0 | 0 |  |  |  |  |  |
|  | 6\| | 0 | 0 | 0 | 0 | 1 |  |  |  |  |
|  | 7\| | 0 | 0 | 1 | 0 | 1 | 1 |  |  |  |
|  | 8\| | 0 | 0 | 0 | 0 | 1 | 1 | 1 |  |  |
|  | 9\| | 0 | 0 | 0 | 0 | 1 | 1 | 1 | 1 |  |
|  | 10\| | 0 | 0 | 0 | 0 | 0 | 0 | 0 | 0 | 1 |
|  |  |  |  |  |  |  |  |  |  |  |
| P9 | 2\| | 1 |  |  |  |  |  |  |  |  |
|  | 3\| | 0 | 0 |  |  |  |  |  |  |  |
|  | 4\| | 0 | 0 | 0 |  |  |  |  |  |  |
|  | 5\| | 1 | 1 | 1 | 0 |  |  |  |  |  |
|  | 6\| | 1 | 0 | 1 | 0 | 1 |  |  |  |  |
|  | 7\| | 1 | 0 | 1 | 0 | 1 | 1 |  |  |  |
|  | 8\| | 1 | 0 | 0 | 0 | 1 | 0 | 0 |  |  |
|  | 9\| | 0 | 0 | 0 | 0 | 0 | 1 | 1 | 0 |  |
|  | 10\| | 1 | 0 | 0 | 0 | 1 | 1 | 1 | 0 | 1 |

| **Table S7** Tests for linkage disequilibrium for 8 *Mytilus galloprovincialis* microsatellites at each of 4 sites. Significance is indicated by a 1, non-significance by a 0. | | | | | | | | | |
| --- | --- | --- | --- | --- | --- | --- | --- | --- | --- |
|  |  | Locus no. | | | | | | | |
| Site |  | 1 | 2 | 3 | 4 | 5 | 6 | 7 | 8 |
| M1 | 2\| | 0 |  |  |  |  |  |  |  |
|  | 3\| | 0 | 0 |  |  |  |  |  |  |
|  | 4\| | 0 | 0 | 0 |  |  |  |  |  |
|  | 5\| | 0 | 0 | 0 | 0 |  |  |  |  |
|  | 6\| | 0 | 0 | 0 | 0 | 0 |  |  |  |
|  | 7\| | 0 | 0 | 0 | 0 | 0 | 0 |  |  |
|  | 8\| | 1 | 0 | 0 | 0 | 0 | 0 | 0 |  |
|  | 9\| | 0 | 0 | 0 | 0 | 0 | 0 | 0 | 0 |
|  |  |  |  |  |  |  |  |  |  |
| M2 | 2\| | 0 |  |  |  |  |  |  |  |
|  | 3\| | 0 | 1 |  |  |  |  |  |  |
|  | 4\| | 0 | 0 | 0 |  |  |  |  |  |
|  | 5\| | 0 | 0 | 0 | 0 |  |  |  |  |
|  | 6\| | 0 | 0 | 0 | 0 | 0 |  |  |  |
|  | 7\| | 0 | 0 | 0 | 0 | 0 | 0 |  |  |
|  | 8\| | 0 | 0 | 0 | 0 | 0 | 0 | 0 |  |
|  | 9\| | 0 | 0 | 0 | 0 | 0 | 0 | 0 | 0 |
|  |  |  |  |  |  |  |  |  |  |
| M3 | 2\| | 0 |  |  |  |  |  |  |  |
|  | 3\| | 0 | 0 |  |  |  |  |  |  |
|  | 4\| | 0 | 0 | 0 |  |  |  |  |  |
|  | 5\| | 0 | 0 | 0 | 0 |  |  |  |  |
|  | 6\| | 0 | 0 | 0 | 0 | 0 |  |  |  |
|  | 7\| | 0 | 0 | 0 | 0 | 0 | 0 |  |  |
|  | 8\| | 0 | 0 | 0 | 0 | 0 | 0 | 0 |  |
|  | 9\| | 0 | 0 | 0 | 0 | 0 | 0 | 0 | 0 |
|  |  |  |  |  |  |  |  |  |  |
| M4 | 2\| | 0 |  |  |  |  |  |  |  |
|  | 3\| | 0 | 0 |  |  |  |  |  |  |
|  | 4\| | 0 | 0 | 0 |  |  |  |  |  |
|  | 5\| | 0 | 0 | 0 | 0 |  |  |  |  |
|  | 6\| | 0 | 0 | 0 | 0 | 0 |  |  |  |
|  | 7\| | 0 | 0 | 0 | 0 | 0 | 0 |  |  |
|  | 8\| | 0 | 0 | 0 | 0 | 0 | 0 | 0 |  |
|  | 9\| | 0 | 0 | 0 | 0 | 0 | 0 | 0 | 0 |

| **Table S8** Tests for departures from Hardy-Weinberg equilibrium for ten *Perna perna* microsatellite loci at each of nine sites. | | | | |
| --- | --- | --- | --- | --- |
| Site | Locus No. | *H*_O_ | *H*_E_ | P |
| P1 | 1 | 0.84783 | 0.86861 | 0.10848 |
|  | 2 | 0.36842 | 0.79659 | 0.00003 |
|  | 3 | 0.68085 | 0.63029 | 0.00383 |
|  | 4 | 0.45652 | 0.70067 | 0.00001 |
|  | 5 | 0.21053 | 0.68807 | 0.00000 |
|  | 6 | 0.17949 | 0.89477 | 0.00000 |
|  | 7 | 0.69767 | 0.94747 | 0.00467 |
|  | 8 | 0.60870 | 0.80339 | 0.00696 |
|  | 9 | 0.33333 | 0.92723 | 0.00000 |
|  | 10 | 0.30435 | 0.59962 | 0.00000 |
|  |  |  |  |  |
| P2 | 1 | 0.87234 | 0.85678 | 0.02894 |
|  | 2 | 0.75000 | 0.8383 | 0.07595 |
|  | 3 | 0.60000 | 0.85468 | 0.00000 |
|  | 4 | 0.3913 | 0.69661 | 0.00001 |
|  | 5 | 0.37778 | 0.91461 | 0.00000 |
|  | 6 | 0.35897 | 0.9041 | 0.00000 |
|  | 7 | 0.39474 | 0.93228 | 0.00000 |
|  | 8 | 0.40000 | 0.86335 | 0.00000 |
|  | 9 | 0.11111 | 0.92173 | 0.00000 |
|  | 10 | 0.45652 | 0.72432 | 0.00000 |
|  |  |  |  |  |
| P3 | 1 | 0.71739 | 0.83899 | 0.0076 |
|  | 2 | 0.92857 | 0.84968 | 0.46101 |
|  | 3 | 0.87234 | 0.84283 | 0.93205 |
|  | 4 | 0.34091 | 0.68365 | 0.00000 |
|  | 5 | 0.30000 | 0.83449 | 0.00000 |
|  | 6 | 0.56818 | 0.92294 | 0.00000 |
|  | 7 | 0.50000 | 0.94593 | 0.00000 |
|  | 8 | 0.34483 | 0.80762 | 0.00000 |
|  | 9 | 0.27778 | 0.93701 | 0.00000 |
|  | 10 | 0.46809 | 0.68749 | 0.00000 |
|  |  |  |  |  |
| P4 | 1 | 0.68182 | 0.81609 | 0.01828 |
|  | 2 | 0.89474 | 0.79263 | 0.75015 |
|  | 3 | 0.77273 | 0.88245 | 0.01768 |
|  | 4 | 0.52500 | 0.67816 | 0.01666 |
|  | 5 | 0.42857 | 0.83312 | 0.00000 |
|  | 6 | 0.45000 | 0.91076 | 0.00000 |
|  | 7 | 0.23333 | 0.95085 | 0.00000 |
|  | 8 | 0.31034 | 0.86449 | 0.00000 |
|  | 9 | 0.47500 | 0.93323 | 0.00000 |
|  | 10 | 0.39535 | 0.60164 | 0.00000 |
|  |  |  |  |  |
| P5 | 1 | 0.42553 | 0.8005 | 0.00000 |
|  | 2 | 0.66667 | 0.64894 | 0.84767 |
|  | 3 | 0.75556 | 0.86617 | 0.20010 |
|  | 4 | 0.61364 | 0.67633 | 0.07047 |
|  | 5 | 0.25926 | 0.9406 | 0.00000 |
|  | 6 | 0.16216 | 0.77971 | 0.00000 |
|  | 7 | 0.64444 | 0.96404 | 0.00009 |
|  | 8 | 0.34286 | 0.80994 | 0.00000 |
|  | 9 | 0.41860 | 0.89029 | 0.00000 |
|  | 10 | 0.25532 | 0.61977 | 0.00000 |
|  |  |  |  |  |
| P6 | 1 | 0.53846 | 0.85248 | 0.00000 |
|  | 2 | 0.82927 | 0.74195 | 0.04643 |
|  | 3 | 0.72727 | 0.9334 | 0.00000 |
|  | 4 | 0.63415 | 0.69527 | 0.31247 |
|  | 5 | 0.11111 | 0.86984 | 0.00000 |
|  | 6 | 0.33333 | 0.91002 | 0.00000 |
|  | 7 | 0.64706 | 0.97498 | 0.00068 |
|  | 8 | 0.37500 | 0.78734 | 0.00000 |
|  | 9 | 0.31250 | 0.95089 | 0.00000 |
|  | 10 | 0.08000 | 0.73306 | 0.00000 |
|  |  |  |  |  |
| P7 | 1 | 0.57447 | 0.78609 | 0.01472 |
|  | 2 | 0.51351 | 0.69345 | 0.02566 |
|  | 3 | 0.77083 | 0.90987 | 0.01901 |
|  | 4 | 0.62500 | 0.7011 | 0.08453 |
|  | 5 | 0.14286 | 0.87338 | 0.00000 |
|  | 6 | 0.41463 | 0.90063 | 0.00000 |
|  | 7 | 0.82927 | 0.98193 | 0.0014 |
|  | 8 | 0.36364 | 0.86024 | 0.00000 |
|  | 9 | 0.21951 | 0.94911 | 0.00000 |
|  | 10 | 0.00000 | 0.68438 | 0.00000 |
|  |  |  |  |  |
| P8 | 1 | 0.68085 | 0.86776 | 0.00185 |
|  | 2 | 0.56522 | 0.54921 | 0.52599 |
|  | 3 | 0.7561 | 0.8931 | 0.00385 |
|  | 4 | 0.6087 | 0.62876 | 0.16828 |
|  | 5 | 0.26667 | 0.88192 | 0.00000 |
|  | 6 | 0.37209 | 0.92695 | 0.00000 |
|  | 7 | 0.65217 | 0.98208 | 0.00000 |
|  | 8 | 0.31818 | 0.79154 | 0.00000 |
|  | 9 | 0.26316 | 0.95298 | 0.00000 |
|  | 10 | 0.00000 | 0.5669 | 0.00000 |
|  |  |  |  |  |
| P9 | 1 | 0.55556 | 0.83271 | 0.00000 |
|  | 2 | 0.69444 | 0.732 | 0.06683 |
|  | 3 | 0.81579 | 0.89404 | 0.30227 |
|  | 4 | 0.60465 | 0.57483 | 0.40247 |
|  | 5 | 0.37500 | 0.90575 | 0.00000 |
|  | 6 | 0.20000 | 0.92848 | 0.00000 |
|  | 7 | 0.53846 | 0.97469 | 0.00076 |
|  | 8 | 0.35714 | 0.81268 | 0.00000 |
|  | 9 | 0.18182 | 0.95245 | 0.00000 |
|  | 10 | 0.00000 | 0.59959 | 0.00000 |

| **Table S9** Tests for departures from Hardy-Weinberg equilibrium for 10 *Mytilus galloprovincialis* microsatellite loci at each of four sites. | | | | |
| --- | --- | --- | --- | --- |
| Site | Locus No. | *H*_O_ | *H*_E_ | P |
| M1 | 1 | 0.5122 | 0.6224 | 0.24242 |
|  | 2 | 0.85417 | 0.7693 | 0.08453 |
|  | 3 | 0.48936 | 0.67216 | 0.01514 |
|  | 4 | 0.20833 | 0.31952 | 0.00386 |
|  | 5 | 0.6875 | 0.78202 | 0.09521 |
|  | 6 | 0.54167 | 0.61535 | 0.46725 |
|  | 7 | 0.58333 | 0.60066 | 0.56928 |
|  | 8 | 0.72917 | 0.89715 | 0.00064 |
|  | 9 | 0.52083 | 0.56513 | 0.00293 |
|  |  |  |  |  |
| M2 | 1 | 0.53191 | 0.52963 | 0.87356 |
|  | 2 | 0.6875 | 0.7432 | 0.32954 |
|  | 3 | 0.33333 | 0.66517 | 0.00000 |
|  | 4 | 0.14894 | 0.2194 | 0.03884 |
|  | 5 | 0.47917 | 0.57566 | 0.54126 |
|  | 6 | 0.50000 | 0.60526 | 0.02108 |
|  | 7 | 0.60417 | 0.6136 | 0.20284 |
|  | 8 | 0.66667 | 0.88509 | 0.00078 |
|  | 9 | 0.41667 | 0.58531 | 0.00291 |
|  |  |  |  |  |
| M3 | 1 | 0.45833 | 0.53991 | 0.39736 |
|  | 2 | 0.72917 | 0.76338 | 0.87909 |
|  | 3 | 0.29787 | 0.65157 | 0.00000 |
|  | 4 | 0.25 | 0.30439 | 0.36479 |
|  | 5 | 0.625 | 0.69254 | 0.15519 |
|  | 6 | 0.64583 | 0.68618 | 0.87677 |
|  | 7 | 0.66667 | 0.5614 | 0.31285 |
|  | 8 | 0.6875 | 0.88947 | 0.00050 |
|  | 9 | 0.39583 | 0.47368 | 0.06170 |
|  |  |  |  |  |
| M4 | 1 | 0.45833 | 0.53991 | 0.39736 |
|  | 2 | 0.72917 | 0.76338 | 0.87909 |
|  | 3 | 0.29787 | 0.65157 | 0.0000 |
|  | 4 | 0.25 | 0.30439 | 0.36479 |
|  | 5 | 0.625 | 0.69254 | 0.15519 |
|  | 6 | 0.64583 | 0.68618 | 0.87677 |
|  | 7 | 0.66667 | 0.5614 | 0.31285 |
|  | 8 | 0.6875 | 0.88947 | 0.00051 |
|  | 9 | 0.39583 | 0.47368 | 0.06170 |

| **Table S10** Pairwise estimates of genetic structure for microsatellite data of *Perna perna*; below diagonal: *F*_ST_ , above diagonal: *G”*_ST_. Bonferroni correction was applied to P-values: *(P<0.05): 0.0056; ** (P<0.01): 0.0011; E: Edge; C: Centre. Bold: pairwise comparisons that were not significant; grey shading: pairwise comparisons of Centre vs. Edge sites; no shading: Centre vs. Centre sites, or Edge vs. Edge sites. | | | | | | | | | |
| --- | --- | --- | --- | --- | --- | --- | --- | --- | --- |
| Site | P1  E | P2  E | P3  C | P4  C | P5  E | P6  E | P7  C | P8  C | P9  C |
| P1 | -- | 0.256** | 0.210** | 0.317** | 0.337** | 0.383** | 0.464** | 0.520** | 0.519** |
| P2 | 0.032** | -- | **0.022** | **0.039** | 0.412** | 0.152** | 0.225** | 0.284** | 0.281** |
| P3 | 0.029** | **0.010** | -- | **0.025** | 0.353** | 0.137** | 0.206** | 0.233** | 0.218** |
| P4 | 0.040** | **0.012** | **0.011** | -- | 0.453** | 0.147** | 0.218** | 0.278** | 0.275** |
| P5 | 0.044** | 0.045** | 0.041** | 0.052** | -- | 0.303** | 0.347** | 0.346** | 0.290** |
| P6 | 0.046** | 0.022** | 0.021** | 0.022** | 0.037** | -- | **0.083** | **0.044** | 0.088* |
| P7 | 0.054** | 0.027** | 0.026** | 0.028** | 0.041** | **0.017** | -- | **0.079*** | **0.070** |
| P8 | 0.064** | 0.033** | 0.030** | 0.035** | 0.044** | **0.014** | 0.016* | -- | **0.027** |
| P9 | 0.062** | 0.032** | 0.028** | 0.034** | 0.037** | 0.018* | **0.015** | **0.012** | -- |

| **Table S11** Pairwise estimates of genetic structure for microsatellite data of *Mytilus galloprovincialis*; below diagonal: *F*_ST_ , above diagonal: *G”*_ST_. Bonferroni correction was applied to P-values: *(P<0.05): 0.0125; ** (P<0.01): 0.0025; E: Edge; C: Centre. Grey shading: pairwise comparisons of Centre vs. Edge sites. | | | | | |
| --- | --- | --- | --- | --- | --- |
|  |  | Centre | | Edge | |
| Category | Site | M1 | M2 | M3 | M4 |
| Centre | M1 | -- | 0.009 | 0.018 | 0.001 |
|  | M2 | 0.008 | -- | 0.028 | 0.013 |
| Edge | M3 | 0.009 | 0.012* | -- | 0.000 |
|  | M4 | 0.007 | 0.009 | 0.004 | -- |

| **Table S12** Pairwise comparisons of a) haplotype diversity *h* and b) nucleotide diversity π for COI sequences of the western lineage of *Perna perna;* below diagonal: difference in diversity index between sites; above diagonal: P-value. These were adjusted for multiple comparisons using Bonferroni correction: corrected P-value for 5 sites at α=0.05: 0.01. Grey shading: pairwise comparisons of Centre vs. Edge sites (Edge West or Edge East). | | | | | | | |
| --- | --- | --- | --- | --- | --- | --- | --- |
|  |  |  | Edge West | | Centre | | Edge East |
| a) | Category | Site | P1 | P2 | P3 | P4 | P5 |
|  | Edge West | P1 | -- | 0.40 | 0.23 | 0.26 | 0.20 |
|  |  | P2 | 0.027 | -- | 0.04 | 0.05 | 0.56 |
|  | Centre | P3 | 0.035 | 0.062 | -- | 0.97 | 0.03 |
|  |  | P4 | 0.034 | 0.061 | 0.001 | -- | 0.04 |
|  | Edge East | P5 | 0.047 | 0.020 | 0.082 | 0.081 | -- |
|  |  |  |  |  |  |  |  |
| b) | Edge West | P1 | -- | 0.64 | 0.21 | 0.50 | 0.71 |
|  |  | P2 | 0.0005 | -- | 0.42 | 0.80 | 0.43 |
|  | Centre | P3 | 0.0013 | 0.0008 | -- | 0.58 | 0.17 |
|  |  | P4 | 0.0007 | 0.0002 | 0.0006 | -- | 0.32 |
|  | Edge East | P5 | 0.0005 | 0.0010 | 0.0018 | 0.0012 | -- |

| **Table S13** Pairwise comparisons of a) haplotype diversity h and b) nucleotide diversity π for COI sequences of the eastern lineage of *Perna perna;* below diagonal: difference in diversity index between sites; above diagonal: P-value. These were adjusted for multiple comparisons using Bonferroni correction: corrected P-value for 5 sites at α=0.05: 0.01. Grey shading: pairwise comparisons of Centre vs. Edge sites. | | | | | | | |
| --- | --- | --- | --- | --- | --- | --- | --- |
|  |  |  | Edge | | Centre | | |
| a) | Category | Site | P5 | P6 | P7 | P8 | P9 |
|  | Edge | P5 | -- | 0.96 | 0.62 | 0.90 | 0.94 |
|  |  | P6 | 0.011 | -- | 0.35 | 0.87 | 0.93 |
|  | Centre | P7 | 0.098 | 0.088 | -- | 0.42 | 0.28 |
|  |  | P8 | 0.027 | 0.017 | 0.071 | -- | 0.94 |
|  |  | P9 | 0.021 | 0.010 | 0.078 | 0.006 | -- |
|  |  |  |  |  |  |  |  |
| b) | Edge | P5 | -- | 0.19 | 0.74 | 0.98 | 0.92 |
|  |  | P6 | 0.0023 | -- | 0.21 | 0.17 | 0.10 |
|  | Centre | P7 | 0.0005 | 0.0018 | -- | 0.61 | 0.65 |
|  |  | P8 | 0.0001 | 0.0024 | 0.0006 | -- | 0.94 |
|  |  | P9 | 0.0002 | 0.0025 | 0.0007 | 0.0001 | -- |

| **Table S14** Maximum and mean distance travelled by the particles advected in the simulation of dispersal for the overall connectivity events and 95th percentile of events. | | | | |
| --- | --- | --- | --- | --- |
|  | Max | Mean | Max (95%) | Mean (95%) |
| Distance | 1002.81 | 59.35±69.86 | 48.02 | 7.48±7.25 |
| Probability | 0.626 | 0.01±0.03 | 0.62 | 0.12±0.06 |
| Time | 30 | 4.97±5.21 | 5.23 | 0.87±0.43 |

| **Table S15** Direct probability of connectivity between locations sampled for genetics averaged for a 10-year period. | | | | | | | | | | |
| --- | --- | --- | --- | --- | --- | --- | --- | --- | --- | --- |
|  | **P1** | **P2** | **P3/M1** | **P4/M2** | **M3** | **P5/M4** | **P6** | **P7** | **P8** | **P9** |
| **P1** | 0 | 0 | 0 | 0 | 0 | 0 | 0 | 0 | 0 | 0 |
| **P2** | 0.00411 | 0 | 0 | 0 | 0 | 0 | 0 | 0 | 0 | 0 |
| **P3/M1** | 0 | 0 | 0 | 0 | 0 | 0 | 0 | 0 | 0 | 0 |
| **P4/M2** | 0 | 0 | 0.00548 | 0 | 0 | 0 | 0 | 0 | 0 | 0 |
| **M3** | 0 | 0 | 0 | 0 | 0 | 0 | 0 | 0 | 0 | 0 |
| **P5/M4** | 0 | 0 | 0 | 0 | 0 | 0 | 0 | 0 | 0 | 0 |
| **P6** | 0 | 0 | 0 | 0 | 0 | 0 | 0 | 0 | 0 | 0 |
| **P7** | 0 | 0 | 0 | 0 | 0 | 0 | 0 | 0 | 0 | 0 |
| **P8** | 0 | 0 | 0 | 0 | 0 | 0 | 0 | 0 | 0 | 0 |
| **P9** | 0 | 0 | 0 | 0 | 0 | 0 | 0 | 0 | 0 | 0 |

| **Table S16** Stepping-stone probability of connectivity between locations sampled for genetics averaged for a 10-year period. | | | | | | | | | | |
| --- | --- | --- | --- | --- | --- | --- | --- | --- | --- | --- |
|  | **P1** | **P2** | **P3/M1** | **P4/M2** | **M3** | **P5/M4** | **P6** | **P7** | **P8** | **P9** |
| **P1** |  | 8,23E-08 | 6,42E-07 | 4,68E-09 | 2,49E-28 | 9,60E-12 | 1,44E-07 | 5,63E-11 | 9,31E-23 | 2,22E-24 |
| **P2** | 4,11E-03 |  | 1,44E-07 | 1,28E-07 | 1,58E-28 | 9,60E-12 | 1,44E-07 | 5,63E-11 | 9,31E-23 | 2,22E-24 |
| **P3/M1** | 1,42E-09 | 1,13E-10 |  | 2,97E-04 | 6,97E-33 | 4,23E-16 | 1,08E-12 | 4,23E-16 | 4,10E-27 | 9,79E-29 |
| **P4/M2** | 5,63E-11 | 3,80E-12 | 5,48E-03 |  | 5,73E-35 | 3,48E-18 | 8,88E-15 | 3,48E-18 | 3,37E-29 | 8,05E-31 |
| **M3** | 2,30E-13 | 1,26E-14 | 4,23E-16 | 2,48E-16 |  | 1,63E-09 | 2,21E-09 | 3,45E-07 | 6,18E-05 | 1,72E-05 |
| **P5/M4** | 2,47E-10 | 1,35E-11 | 3,33E-12 | 2,66E-13 | 5,33E-21 |  | 3,51E-06 | 6,91E-05 | 1,57E-15 | 3,75E-17 |
| **P6** | 3,31E-09 | 2,63E-10 | 9,80E-12 | 7,23E-13 | 1,24E-22 | 7,51E-06 |  | 9,42E-07 | 7,28E-17 | 1,74E-18 |
| **P7** | 1,03E-10 | 5,60E-12 | 2,10E-12 | 1,11E-13 | 1,47E-19 | 3,30E-05 | 8,69E-07 |  | 2,66E-14 | 6,34E-16 |
| **P8** | 1,13E-11 | 6,18E-13 | 1,64E-14 | 1,22E-14 | 1,94E-08 | 2,06E-08 | 2,51E-07 | 1,01E-06 |  | 4,80E-05 |
| **P9** | 4,31E-11 | 2,35E-12 | 6,23E-14 | 4,64E-14 | 1,50E-05 | 4,85E-09 | 4,52E-07 | 1,16E-07 | 3,87E-04 |  |

**Mov S1** Simulation of potential dispersal for marine mussels in South Africa running for the year 2012. Random release locations (in different colours) were chosen to better illustrate the path of individual particles. Link: <https://figshare.com/s/b8148f12e2a2cffae907>; DOI: 10.6084/m9.figshare.8910812

**Supplementary references**

Presa P, Pérez M, Diz AP (2002) Polymorphic microsatellite markers for blue mussels (*Mytilus* spp.). Conserv Genet 3:441–443. doi: 10.1023/A:1020571202907

Varela MA, González-Tizón A, Mariñas L, Martínez-Lage A (2007) Genetic divergence detected by ISSR markers and characterization of microsatellite regions in *Mytilus* mussels. Biochem Genet 45:565–578. doi: 10.1007/s10528-007-9097-7

Vera M, Pardo BG, Pino‐Querido A, Álvarez‐Dios JA, Fuentes J, Martínez P (2010) Characterization of single-nucleotide polymorphism markers in the Mediterranean mussel, *Mytilus galloprovincialis*. Aquac Res 41:e568–e575. doi: 10.1111/j.1365-2109.2010.02550.x

Yu H, Li Q (2007) Development of EST-SSRs in the Mediterranean blue mussel, *Mytilus galloproviancialis*. Mol Ecol Notes 7:1308–1310. doi: 10.1111/j.1471-8286.2007.01865.x
